# Designing antiviral surfaces to suppress the spread of COVID-19

**DOI:** 10.1063/5.0049404

**Published:** 2021-05-04

**Authors:** Sanghamitro Chatterjee, Janani Srree Murallidharan, Amit Agrawal, Rajneesh Bhardwaj

**Affiliations:** Department of Mechanical Engineering, Indian Institute of Technology Bombay, Mumbai 400076, India

## Abstract

Surface engineering is an emerging technology to design antiviral surfaces, especially in the wake of COVID-19 pandemic. However, there is yet no general understanding of the rules and optimized conditions governing the virucidal properties of engineered surfaces. The understanding is crucial for designing antiviral surfaces. Previous studies reported that the drying time of a residual thin-film after the evaporation of a bulk respiratory droplet on a smooth surface correlates with the coronavirus survival time. Recently, we [Chatterjee *et al.*, Phys. Fluids. **33**, 021701 (2021)] showed that the evaporation is much faster on porous than impermeable surfaces, making the porous surfaces lesser susceptible to virus survival. The faster evaporation on porous surfaces was attributed to an enhanced disjoining pressure within the thin-film due the presence of horizontally oriented fibers and void spaces. Motivated by this, we explore herein the disjoining pressure-driven thin-film evaporation mechanism and thereby the virucidal properties of engineered surfaces with varied wettability and texture. A generic model is developed which agrees qualitatively well with the previous virus titer measurements on nanostructured surfaces. Thereafter, we design model surfaces and report the optimized conditions for roughness and wettability to achieve the most prominent virucidal effect. We have deciphered that the optimized thin-film lifetime can be gained by tailoring wettability and roughness, irrespective of the nature of texture geometry. The present study expands the applicability of the process and demonstrates ways to design antiviral surfaces, thereby aiding to mitigate the spread of COVID-19.

## I. INTRODUCTION

The ongoing COVID-19 pandemic caused by the SARS-CoV-2 (referred to as coronavirus hereafter) has created a huge health and an economic crisis throughout the world. The disease spreads via respiratory droplets, a fact which is well-documented.^1–11^ Researchers have devoted significant attention in investigating and analyzing the different routes of disease transmission and their relative importance.^12^ The governing transport mechanism for the virus to attack a target is its rotational diffusivity.^13^ The spread of infection can be airborne, i.e., via aerosol, and the persistence of the aerosols plays a significant role in determining the transmission probability.^14–20^ Apart from airborne transmission, the virus-laden droplets can also deposit on a surface forming fomite, which serves as secondary source of transmission upon touch.^21,22^ Several measures to mitigate the disease transmission have been studied and demonstrated by the researchers across the globe. Face masks and face shields have been found to be the most effective ways to stop the disease spread through the aerosol route of transmission.^23–30^ To reduce the risk of infection through fomite route, sanitization of surfaces was recommended by the WHO.^31^ In addition, Chen *et al.*^32^ demonstrated a new way of deactivating coronavirus by applying cold atmospheric plasma (CAP). However, in many circumstances, it may be inconvenient to disinfect a contaminated surface by frequent sanitization/CAP processing. This fact marks the need to fabricate surfaces with *virucidal properties*, i.e., surface properties by virtue of which the virus cannot survive longer on them and the deposited viral load decays rapidly.^33,34^ For example, it was previously demonstrated that polycations of polymer surfaces cause rapid viral disintegration.^35,36^ However, the applicability of such coating technology is limited by lack of durability and less mechanical stability, which demands design of more robust antiviral surfaces.^33,34,37^

Since the aqueous phase of the respiratory droplet serves as a medium for survival of enveloped virus such as coronavirus, the dynamics of the droplet plays an important role in deciding the transmission probability. In particular, evaporation determines the eventual fate of the droplet, and thereby it is correlated with the survival timescale of the virus.^38–40^ It has been demonstrated that the time decay of virion concentration is correlated with the volume loss of the respiratory droplet due to evaporation.^41^ For this reason, the virus survival and infection spread are related to the environmental condition, e.g., ambient temperature and relative humidity.^1,42,43^ To this extent, recently, Bhardwaj and Agrawal^44,45^ established that by considering a surrogate droplet of pure water, the drying timescale of the droplet and a residual thin film after the evaporation of the bulk droplet are correlated with the decay timescale of virus titer on different surfaces. While the evaporation of the bulk droplet is much faster [∼O(s)], which is governed by the diffusion of liquid–vapor (LV) outside the droplet,^44^ the evaporation of the residual thin film is governed by the disjoining pressure and is a much slower process than the former.^45^ Therefore, the lifetime of the residual thin-film contributes to the maximum portion of virus survival time, and attention should be devoted to look at the evaporation rate of the residual thin-film to analyze the virus survival time on the surface in question. More recently, Chatterjee *et al.*^46^ demonstrated that the evaporation rate of the residual thin-film is much faster on porous surfaces than impermeable surfaces, making the porous media lesser susceptible to virus survival. The aforesaid faster evaporation rate was attributed to the modification of the effective solid–liquid (SL) interfacial area due to the presence of horizontally oriented fibers and void spaces on porous surfaces, which leads to an enhancement in the energy required to form unit area solid–liquid interface, and thereby resulting in an augmented disjoining pressure within the thin-film.

Motivated by the aforementioned facts, the authors of the present manuscript analyze the drying of a respiratory droplet and residual thin-film on physically textured surface with varying wettability. In particular, it is imperative to explore the effect of surface texturing on the effective solid–liquid interfacial area, and thereby the resultant thin-film evaporation rate. On the other hand, wettability of the underlying surface would additionally contribute to the thin-film evaporation rate by virtue of its influence on the excess energy within the film.^45^ Tailoring wettability was previously found to be a promising tool for curbing the risk of infection;^47^ however, its coupling with surface texture remains unknown. The motivation further arises from the fact that surface modification and engineering are learnt to be a contemporary and yet emerging technology to achieve antiviral surfaces.^33,34^ While there have been several previous efforts to design antibacterial surfaces by surface engineering,^48,49^ Hasan *et al.*^50^ made a valuable contribution in this direction by fabricating nanostructured surfaces by wet-etching to induce *antiviral* properties, and they monitored the virus titer at different times after the surfaces were exposed to viral inoculum. They found that the structured surfaces are much lesser conducive to virus survival as compared to flat surfaces. However, their study was limited to aluminum surfaces, and how one can control the texture to achieve optimized virucidal effects remained an unanswered question.^33^ In view of the above, the distinction of the present study is that it aims at answering the following specific research questions. (1) What is the combined effect of wettability and surface texture on the evaporation dynamics of the residual thin-film? The insights would be useful in advancing the fundamental knowledge in the field. This is because the physics of thin-liquid films on solid surfaces has engaged the researchers to study variety of phenomena,^51^ e.g., formation of films,^52,53^ stability or instability of the thin-film under different conditions,^54–56^ the effect of surface heterogeneity on the instability and pattern of thin-films,^57^ and subsequent dewetting mechanism and patter formations,^58–60^ and their molecular origin has been extensively studied both numerically^61^ and by experiments.^62^ Thus, the thin-film evaporation mechanism on textured surfaces and understanding the factors governing it are fundamental research questions. (2) From the COVID-19 point of view, is there any optimum condition of wettability and surface roughness in which the drying rate is the highest? (3) How one can tailor these factors in order to achieve the maximum virucidal effects? Finding answers of the above research questions would have important implications. This is because our previous study^46^ demonstrated that the impermeable materials are more susceptible to coronavirus survival than the porous materials. Thereby, the present study is an attempt to tailor the impermeable surfaces for making them lesser conducive to virus survival.

## II. THEORY

### A. Background

**FIG. 1.**
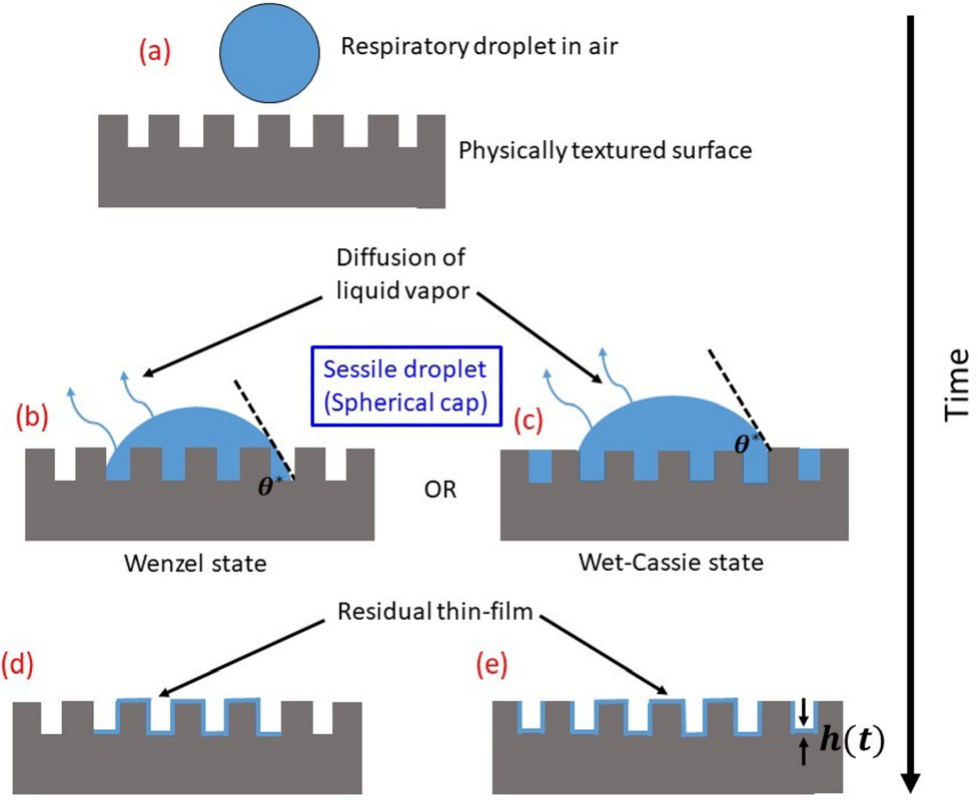
Schematic of the problem considered in the present work.

With an aim to expand the applicability of the process, we first develop a generic analytical model to understand the thin-film evaporation rate in relation to the virucidal properties of textured surfaces, based upon the knowledge gained from our previous study.^45,46^ The model is first compared with the previously reported virus titer measurements,^50^ and a reasonable agreement has been found (cf. Sec. III). Thereafter, we demonstrate model surfaces with varied texture and wettability, and the optimized condition for achieving the desired virucidal effects has been analyzed (cf. Sec. IV). Figure 1 depicts the schematic diagram of the problem. A respiratory droplet is first deposited on a hydrophilic, textured surface [cf. Fig. 1(a)]. Depending upon the roughness and intrinsic wettability of the surfaces, the droplet can remain either in Wenzel or in Wet–Cassie regime (discussed later in Sec. IV A). After deposition, the droplet first undergoes diffusion-limited evaporation in either state [cf. Figs. 1(b) and 1(c)]. Thereafter, a thin residual film is left behind the evaporated droplet. The formation of the residual thin-film would depend on the regime in which the previously deposited droplet remained [cf. Figs. 1(d) and 1(e)]. The different regimes and conditions for their applicability will be discussed below in line with the model formulation and results.

### B. Model for thin-liquid film evaporation on engineered surface

We develop an analytical model for the evaporation mechanism of the thin-liquid film, which remains after the diffusion-limited evaporation of the bulk droplet, as shown schematically in Figs. 1(d) and 1(e). The time variation of thickness *h*(*t*) [cf. Figs. 1(d) and 1(e)] of the evaporating thin film on a smooth solid surface is given by^45^

$$\frac{dh}{dt}=\frac{J}{\rho_{L}},$$ (1)

where *ρ_L_* is the liquid density (=1000 kg/m^3^, for water), and the evaporation mass flux *J* is given by^45^

$$J=\frac{\rho_{V}}{\rho_{L}\sqrt{2\pi rT_{\mathrm{amb}}}}\left[\frac{A_{H}}{6\pi h^{3}}-\frac{\gamma h(t)}{R^{2}}\right],$$ (2)

where *A_H_*, *γ*, and *R* are, respectively, the Hamaker constant of interaction between liquid–vapor and solid–liquid interfaces, the surface tension of the liquid (0.072 N/m, for water), and wetted radius. In Eq. (2), r = 461.5 J/kg K is the specific gas constant for water vapor, *ρ_V_* = 0.023 kg/m^3^ is the density of water vapor at ambient, and *T_amb_* = 298 K is the ambient temperature. Using the values of r, *ρ_V_*, and *T_amb_*, the prefactor outside the parenthesis of Eq. (2) can be calculated as follows: $a=2.47\times 10^{-11}$ SI units. The first term within the parenthesis of Eq. (2) represents the disjoining pressure *P*(*h*) within the film, while the second term is the Laplace pressure. It was previously shown^45,46^ that the Laplace pressure is one order of magnitude less than the disjoining pressure for nanometric thin-films and, therefore, can be ignored in Eq. (2). Hence, neglecting the Laplace pressure term in Eq. (2) and then integrating Eq. (1) with respect to time (*t*) give *h*(*t*) as a function of *t* as follows:^46^

$$h^{4}(t)=h_{0}^{4}+\frac{4aA_{H}}{6\pi }t.$$ (3)

Next, the effect of surface modification (both in terms of wettability and texture) on the evaporation mechanism of the residual thin-film will be formulated. The equilibrium between the different interfacial energies, namely, the liquid–vapor (*γ_LV_*), solid–vapor (*γ_SV_*), and solid–liquid (*γ_SL_*) interfacial energies in terms of contact angle (*θ*), is given by the classical Young's equation^63,64^

$$\gamma_{LV}\;\text{cos}\;\theta =(\gamma_{SV}-\gamma_{SL})=E_{SL},$$ (4)

where *E_SL_* is the energy required to form a unit area of solid–liquid interface.^65^ On the other hand, for the case of a film covered surface, the modified surface energy ($\gamma_{SV}^{\prime }$) reads as^64,66^

$$\gamma_{SV}^{\prime }=\gamma_{SL}+\gamma_{LV}+e(h),$$ (5)

where e(h) is the excess energy of the film, which is the derivative of the disjoining pressure ($P(h)=A_{H}/6\pi h^{3}(t)$) within the thin-film. Considering that for a nanometric thin-film *γ_LV_* is one order magnitude less than e(h); if the quantity $E_{SL}=(\gamma_{SV}^{\prime }-\gamma_{SL})$ is enhanced by a factor *λ*, then e(h) would also be enhanced by the same factor. With this enhancement, the modified evaporation mass flux (*J*) becomes [cf. Eqs. (1) and (2)] $J_{\mathrm{mod}}=\lambda J$. If a surface processing technique (including both chemical modification and physical texture) modifies the apparent contact angle of the surface^63,64^ in question from *θ*_0_ to $\theta^{*}$, then it directly follows from Eq. (4) that

$$\lambda =\frac{\;\text{cos}\;\theta^{*}}{\;\text{cos}\;\theta_{0}}.$$ (6)

Hence, if $\theta^{*}$ and *θ*_0_ are known, *λ* and the corresponding *J_mod_* can be obtained to evaluate the modified *h*(*t*) from Eq. (3). Therefore, the enhancement of a microscopic quantity e(h) [or *P*(*h*)] can be obtained from macroscopic contact angle values.

In the framework of the formulation, the effect of surface wettability and texture on the resultant *λ* can be separately discerned. The contribution of wettability can be separated by evaluating the Hamamker contact (*A_H_*) of interaction between the *SL* and *LV* interfaces for the corresponding contact angle. In a process involving both chemical modification and physical modification by texture-induced roughness of a surface, let us assume that the change in contact angle and associated *A_H_* are as follows: $\theta_{0}\overset{\mathrm{chemical}}{\to }\theta_{i}\overset{\mathrm{texture}}{\to }\theta^{*}$, wherein $\theta_{0}\overset{\mathrm{chemical}}{\to }\theta_{i}\Rightarrow {\left[A_{H}\right]}_{\theta_{0}}\to {\left[A_{H}\right]}_{\theta_{i}}$. Depending upon the intrinsic wettability and the roughness, the droplet-substrate system may remain either in Wenzel or Wet–Cassie state (cf. Fig. 1),^63,64^ which, along with wettability, would determine the resultant *λ* and thereby the formation of the residual thin-film. The details of these states, their applicability, and conditions along with the incorporation in the present model will be discussed later (cf. Sec. IV A).

We first evaluate the Hamaker constant corresponding to the *SL* and *LV* interfacial interaction from the contact angle. To evaluate ${\left[A_{H}\right]}_{\theta_{0}}$ or ${\left[A_{H}\right]}_{\theta_{i}}$, we consider a generic contact angle *θ* to obtain a generic *A_H_*.^65–67^ The solid–liquid adhesive energy (*W_SL_*) for some *θ* reads as

$$W_{SL}=\gamma_{LV}(1+\text{cos}\;\theta ),$$ (7)

where $\gamma_{LV}=0.072$ N/m for water. Since the dispersive component of the surface interaction energies dominates wetting phenomena,^65^ *W_SL_* is related to dispersive component of the surface free energy of the solid ($\gamma_{SS}^{d}$) and liquid ($\gamma_{LL}^{d}=0.022$ N/m for water^65^) by the Berthelot's geometric mean rule^67^ which reads as

$$W_{SL}=2\sqrt{\gamma_{SS}^{d}\gamma_{LL}^{d}}.$$ (8)

The Hamaker constant of the solid surface in question (*A*_11_) is related to ($\gamma_{SS}^{d}$) as^68^

$$\gamma_{SS}^{d}=\frac{A_{11}}{24\pi D_{0}^{2}},$$ (9)

where *D*_0_ is the interfacial contact separation, and an “universal constant value” of 0.165 nm can be assigned to it.^65,66,68^ From Eqs. (7)–(9), *A*_11_ can be obtained from *θ*. The Hamaker constant *A_H_* of interaction between *SL* and *LV* interfaces can be evaluated from *A*_11_, and the Hamaker constant of air ($A_{22}=0$) and liquid ($A_{33}=3.7\times 10^{-20}$ J) can be evaluated by using the following relation:^46,68^

$$A_{H}=(\sqrt{A_{11}}-\sqrt{A_{33}})(\sqrt{A_{22}}-\sqrt{A_{33}}).$$ (10)

Plugging *A_H_* computed from Eq. (10) and *λ* from Eq. (6), we obtain *J_mod_* and corresponding *h*(*t*) for an engineered surface by using Eq. (3).

## III. VALIDATION AGAINST PREVIOUS SARS-CoV-2 TITER MEASUREMENTS

In our previous reports,^45,46^ the change in the thickness of the liquid film with time and its correlation with the slope of reduction of coronavirus titer with time was examined. The slope of reduction of virus titer was found to match qualitatively well with the thickness (or volume) change with time of the film for all cases examined in the studies. The thin-film thickness variation explained the coronavirus survival both for the cases of impermeable and porous surfaces. Thus, the analysis yields useful results to explain the virus survival time on different surfaces qualitatively; that the time-varying virus titer scales with the time-varying thin-film thickness, and thereby assessing the risk factors associated with different surfaces of use. Motivated by these findings, herein we compare the model developed to obtain the time-varying thin-film thickness *h*(*t*) on textured surfaces (cf. Sec. II B) with the previous virus titer measurements on nanostructured surfaces.^50^ In the previous virus titer measurements,^50^ pure aluminum (Al 6063 alloy) substrate and nanostructured aluminum surface fabricated by wet-etching (called as “etched aluminum” surface) were exposed to 10 *μ*l of viral inoculum ($∼10^{5}$ TCID_50_/ml), and the virus titer [TCID_50_/ml (log_10_)] was monitored at different time points after the exposure.^50^ They reported that the wet-etching process leads to the formation of aluminum hydroxide and nanostructures grouped as ridges. This process alters the apparent contact angle from $\theta_{0}=96.3{}^{\circ}$ to $\theta^{*}=17.7{}^{\circ}$.^50^ Following the formulation presented in Sec. II B, we get ${\left[A_{H}\right]}_{\theta_{0}}=3.1\times 10^{-20}$ J. Using this value for *A_H_*, and *λ* = 1 and 8.4155 [cf. Eq. (6)] for smooth and etched aluminum surfaces, respectively, one can evaluate the time evolution of residual thin-film thickness [*h*(*t*)] on them. This analysis takes into account both the effect of chemical and roughness modification induced by the wet-etching process, enabling us to compare the virucidal properties of etched aluminum and smooth aluminum surfaces.

**FIG. 2.**
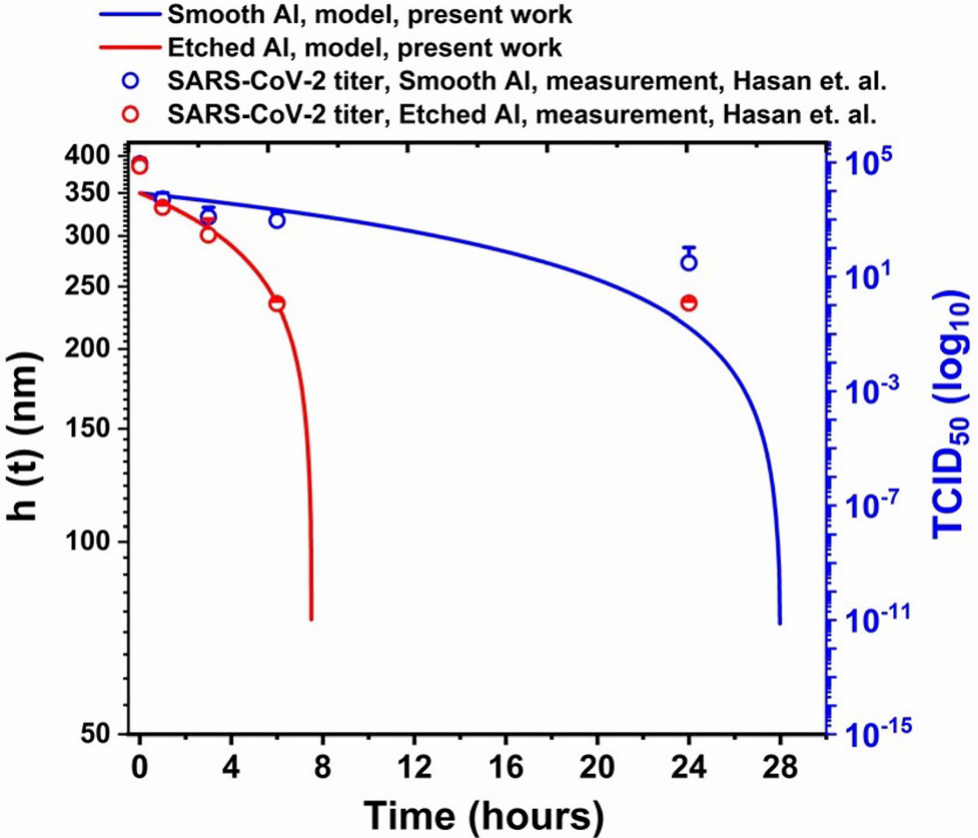
Time-varying evaporating thin-film thickness (*h*, plotted as blue and red lines for smooth and etched aluminum surfaces, respectively) and virus titer [TCID_50_(log_10_), plotted as blue and red circles for smooth and etched aluminum surfaces, respectively]. The virus titer data with the error bar have been reproduced from the recent study.^50^ The initial thin-film thickness is taken as 350 nm.

Figure 2 shows a qualitative comparison of time-varying thin-film thickness with the previous virus titer measurements.^50^ The initial film thickness is taken as 350 nm in the present calculations. The slope of the time-varying film thickness agrees qualitatively with the virus titer decay with respect to time, with comparable thin-film lifetime and virus survival time on smooth and etched aluminum surfaces. On smooth aluminum surfaces, 3–4 log reduction (∼99.9%−99.99%) in the virus titer was recorded after 24 h of exposure^50^ (cf. Fig. 2). The present model predicts an equivalent decay in thin-film thickness within ∼28 h. On etched aluminum surfaces, the virus was effectively inactivated within 6 h of exposure (5-log reduction),^50^ and from Fig. 2, it is noted that the model also returns the same timescale (∼8 h) of decay of the liquid-thin film thickness due to the disjoining pressure-driven evaporation. Overall, the time-varying thin-film thickness matches well with the decay of virus titer reported earlier,^50^ and the agreement between the two dataset is consistent with our previous paper.^45,46^ Thus, the analytical model captured the higher virucidal effect of the etched aluminum surface as compared to smooth aluminum surface. It is noteworthy that the present model considers the enhancement of thin-film evaporation rate (and hence the virucidal property) by virtue of contact angle modification, which is a macroscopic and easily measurable quantity. Furthermore, since the contact angle modification is the result of both chemical modification and texture induced roughness, the model incorporates both the effects by a single parameter. Therefore, a generic model has been developed, irrespective of the specific feature of the surface geometry, which may help in expanding the applicability of the process of surface modification and texturing to induce virucidal effects.

## IV. DESIGNING ANTIVIRAL SURFACES

### A. Concept

The above analysis and comparison with virus titer data motivate us to model engineered surfaces across a wide variation of wettability and texture induced roughness. For this, a few physical aspects have been considered. First, it is well-known that if the surface under question is *intrinsically* hydrophobic ($\theta_{0}>90{}^{\circ}$ or $\text{cos}\;\theta_{0}<1$), then $\theta^{*}>\theta_{0}$ (or $\text{cos}\;\theta^{*}<\text{cos}\;\theta_{0}$).^63,64^ Hence, roughening a surface with $\theta_{0}>90{}^{\circ}$ would reduce *λ* by virtue of Eq. (6), and therefore, the thin-film evaporation would be decelerated resulting in a longer virus survival time. Therefore, a surface engineering process should first modify the contact angle such that $\theta_{i}<90{}^{\circ}$.

In the process exercised by Hasan *et al.*,^50^ this criterion was met by the formation of aluminum hydroxide due to wet-etching, confirmed by energy dispersive x-ray spectroscopy (EDS) and x-ray photoelectron spectroscopy (XPS) measurements. Once a $\theta_{i}<90{}^{\circ}$ is obtained, surface texture can further lead to a $\theta^{*}<\theta_{i}$, as it is well-documented that roughness enhances the hydrophilicity of an intrinsically hydrophilic surface.^63,64^ Therefore, in the analysis presented below for the model surfaces, *θ_i_* is varied in the range of 0° to 90°, and the quantity $\lambda_{r}=\text{cos}\;\theta^{*}/\;\text{cos}\;\theta_{i}$ is defined as the contribution of physical texture induced roughness to *λ* in Eq. (6). A rough surface is characterized by two factors: (i) the roughness factor, *r*, which is the ratio between the actual area of the rough surface and its projected area and (ii) the solid area fraction, $\phi_{s}$, which is the ratio between the solid area at the top surface and the projected area. Furthermore, when a droplet is deposited on a rough surface with $\theta_{i}<90{}^{\circ}$, two distinct regimes^63,64^ are possible depending upon the intrinsic wettability (characterized by *θ_i_*) and the roughness (characterized by *r* and $\phi_{s}$), as shown schematically in Fig. 1. The first regime is the Wenzel regime, realized for higher *θ_i_*, in which the droplet triple phase contact line follows all the topographical variations of the surface [cf. Fig. 1(b)]. This regime is characterized by $\text{cos}\;\theta^{*}=r\;\text{cos}\;\theta_{i}$. Hence, in the Wenzel regime, $\lambda_{r}=r$. However, there exists a limit in the applicability of the Wenzel formulation that a surface cannot be made infinitely hydrophilic by inducing roughness, which precipitates the onset of the second regime discussed below.

The second regime is the hemiwicking (henceforth, referred to as Wet–Cassie) regime, in which a part of the liquid departs from the droplet and impregnates through the crevices, and the rest of the droplet resides on a patchwork of solid and liquid, as shown schematically in Fig. 1(c). This regime is characterized by $\text{cos}\;\theta^{*}=\phi_{s}\;\text{cos}\;\theta_{i}+(1-\phi_{s})$. Hence, in this regime, $\lambda_{r}=\phi_{s}+\frac{1-\phi_{s}}{\;\text{cos}\;\theta_{i}}$. In either regime, $\theta^{*}$ satisfies the condition that $0{}^{\circ}\leq \theta^{*}\leq 90{}^{\circ}$, i.e., $0\leq \text{cos}\;\theta^{*}\leq 1$, and it should be noted that the Wet–Cassie regime is characterized by a lesser enhancement in the hydrophilicity,^63,64^ i.e., lesser enhancement in the energy required to form unit area of solid–liquid interface.

The condition for realizing the Wet–Cassie regime is that *θ_i_* must be less than a critical value, *θ_c_*, such that $\text{cos}\;\theta_{c}=\frac{1-\phi_{s}}{r-\phi s}$. Hence, whether for a given surface, the transition from Wenzel to Wet–Cassie regime is determined by both the chemical details (by virtue of *θ_i_*) as well as the geometric features (by virtue *θ_c_*). Due to the differences in the droplet interaction with the substrate surface in the aforesaid two regimes, the formation of the residual thin film after the diffusion-limited evaporation of the bulk droplet would also be different, as schematically shown in Figs. 1(d) and 1(e). The modified evaporation mass flux (*J_mod_*) of the residual thin-film will, thus, be governed by the appropriate *λ_r_* and ${\left[A_{H}\right]}_{\theta_{i}}$, thereby modifying Eq. (3) as

$$h^{4}=h_{0}^{4}+\frac{4a\lambda_{r}{\left[A_{H}\right]}_{\theta_{i}}}{6\pi }t.$$ (11)

The factor $\lambda_{r}{\left[A_{H}\right]}_{\theta_{i}}$ is, therefore, crucial in dictating the temporal variation of film thickness *h* (hence, the film evaporation rate) on the engineered surfaces. Below, we analyze the optimum conditions for *θ_i_* and *r* to achieve the fastest evaporation rate of the residual thin-film, leading to the most effective virucidal properties.

### B. Proposed design

**FIG. 3.**
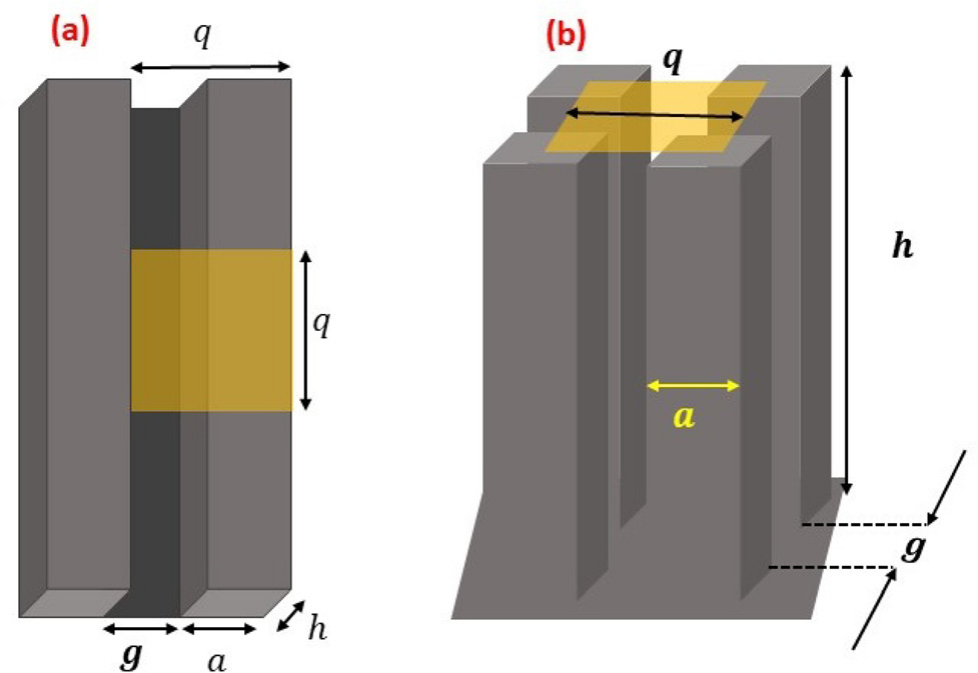
Schematic of the model surfaces: (a) with rectangular parallel grooves and (b) with rectangular pillars. *h*: surface height with respect to the base; *a*: lateral dimension of the heights; *g*: gap between the heights; and q=a+g: pitch.

We consider two engineered surfaces, which are shown schematically in Fig. 3: (i) surfaces with rectangular parallel grooves [cf. Fig. 3(a)] and (ii) surfaces with rectangular pillars [cf. Fig. 3(b)], as it was previously demonstrated that an arbitrary rough surface can be well-approximated by a square-wave generic model in two dimensions to estimate *r* and $\phi_{s}$^69^ by virtue of which the model detailed in Sec. II B can be applied. These simpler geometries are easier to model and from a practical point of view, such surfaces are easier to fabricate by conventional, widespread nano/micromachining techniques, such as focused ion beam (FIB), electron beam lithography, and photo lithography.^70^ The generic model that was employed to examine the correlation between the thin-film lifetime and the virus survival time in Sec. III will be applied herein to analyze the model surface configurations depicted in Fig. 3. This way, the two model surfaces considered herein, is sufficient to reach the research goal, as highlighted in Sec. I.

As shown in Fig. 3, for both surfaces, the lateral dimensions of the features of height *h* (with respect to the base) are *a *×* a*, having a gap of *g* between them. Hence, the pitch is q=a+g. From geometry, *r* and $\phi_{s}$ are determined as^71^

$$r=1+\frac{2h/a}{\left(1+g/a\right)}$$ (12)

and

$$\phi_{s}=\frac{1}{\left(1+g/a\right)}$$ (13)

for surfaces with rectangular grooves; and

$$r=1+\frac{4h/a}{{\left(1+g/a\right)}^{2}}$$ (14)

and

$$\phi_{s}=\frac{1}{{\left(1+g/a\right)}^{2}}$$ (15)

for surfaces decorated with rectangular pillars.

### C. Analysis and optimization of parameters

**FIG. 4.**
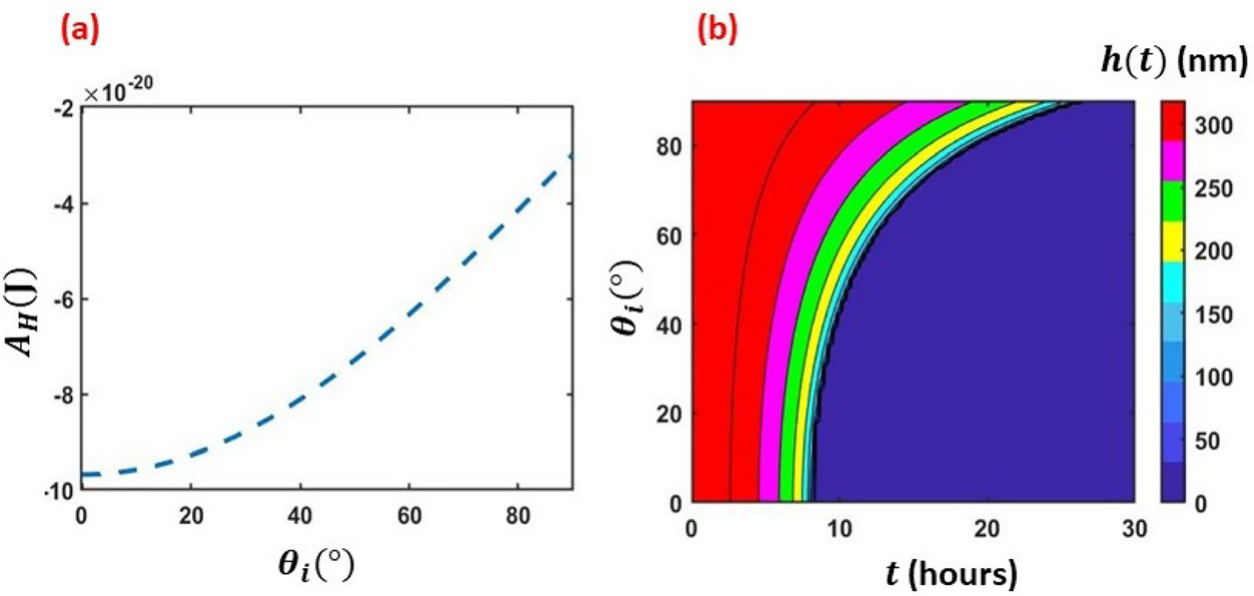
Analysis of thin-film evaporation on *smooth* surface; (a) variation *A_h_* with *θ_i_* and (b) regime map of film thickness *h*(*t*) with respect to time (*t*) and *θ_i_*.

Next, we analyze the thin film evaporation mechanism on the model surfaces for $\theta_{i}=[0{}^{\circ},90{}^{\circ}]$. However, for the sake of contrast and comparison, first a smooth surface ($\lambda_{r}=1$) is considered. From Fig. 4, it is seen that $\left|A_{H}\right|$ increases with decreasing *θ_i_* [cf. Eqs. (7)–(10)]. Consistent with our earlier findings,^45^ the thin-film lifetime is lesser for lower *θ_i_* (∼25 h for $\theta_{i}=90{}^{\circ}$ to ∼8 h for $\theta_{i}=0{}^{\circ}$). Therefore, our present analysis meets the physical requirements.

**FIG. 5.**
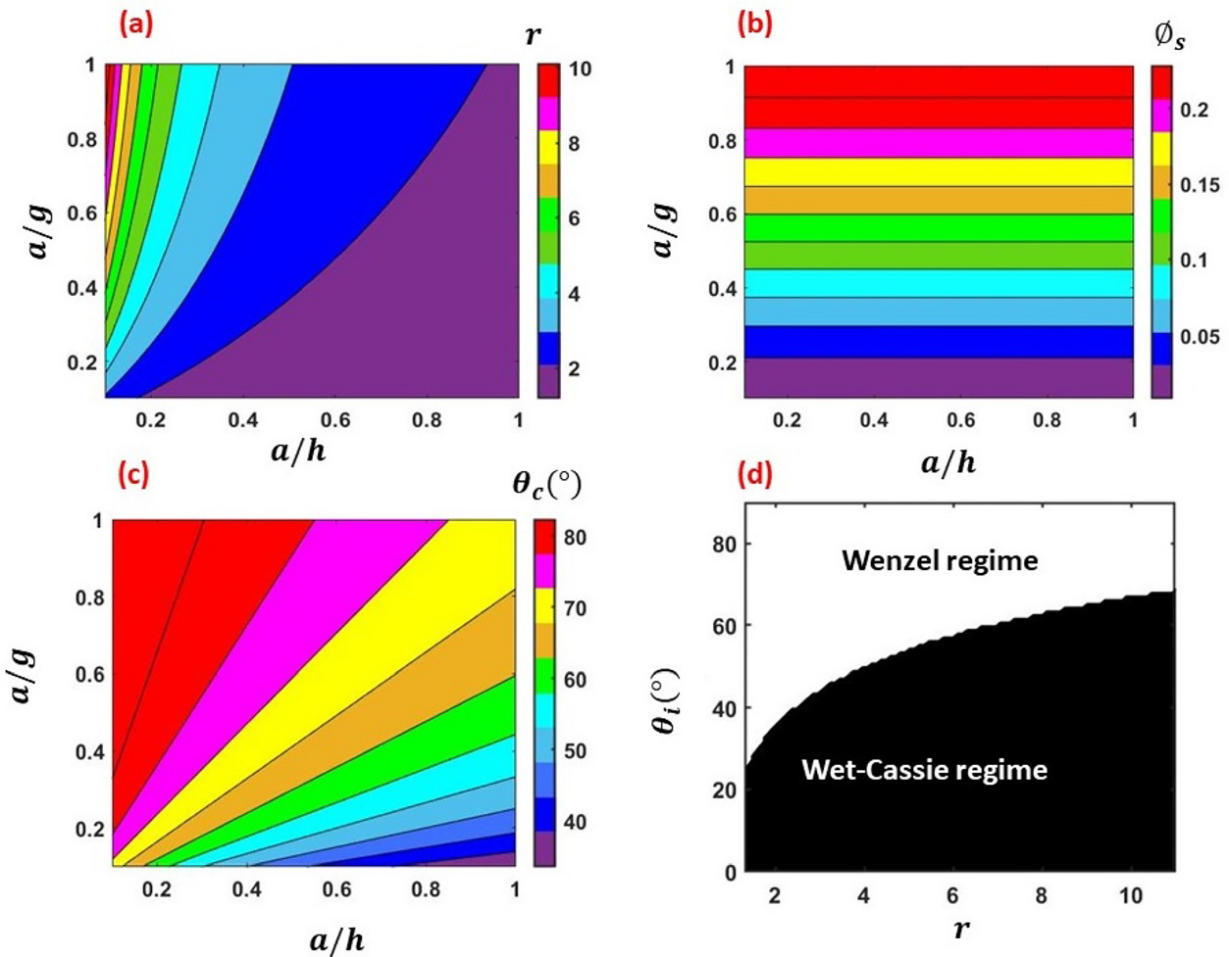
(a) Variation of *r* with respect to *a*/*h* and *a*/*g*, (b) variation of $\phi_{s}$ with respect to *a*/*h* and *a*/*g*, (c) regime map of *θ_c_* for varying *a*/*h* and *a*/*g*, and (d) the Wenzel and Wet–Cassie regimes for varying *θ_i_* and *r*. Results shown for the *grooved* surfaces.

Furthermore, we analyze the engineered surfaces with rectangular parallel grooves [cf. Fig. 3(a)]. Figures 5(a) and 5(b) show the regime maps of *r* and $\phi_{s}$ with respect to *a*/*h* and *a*/*g* for the model grooved surfaces. As expected, *r* increases for low *a*/*h* and high *a*/*g*, and $\phi_{s}$ is independent of *a*/*h*, however, increases with *a*/*g*. Using these regimes for *r* and $\phi_{s}$, we attempt to compute *θ_c_*, and thereby, the regimes for the applicability of Wenzel and Wet–Cassie formulation for the model grooved surfaces are deciphered. From Fig. 5(c), it is seen that for the model grooved surfaces, *θ_c_* varies from ∼25° to ∼80° within the range of *a*/*h* and *a*/*g* considered herein. Accordingly, Fig. 5(d) depicts that the system would remain in Wenzel regime for higher *θ_i_* and in the Wet–Cassie regime for low *θ_i_*. For low roughness, lesser *θ_i_* is required for transition from Wenzel to Wet–Cassie regime. The opposite is true for higher roughness.

**FIG. 6.**
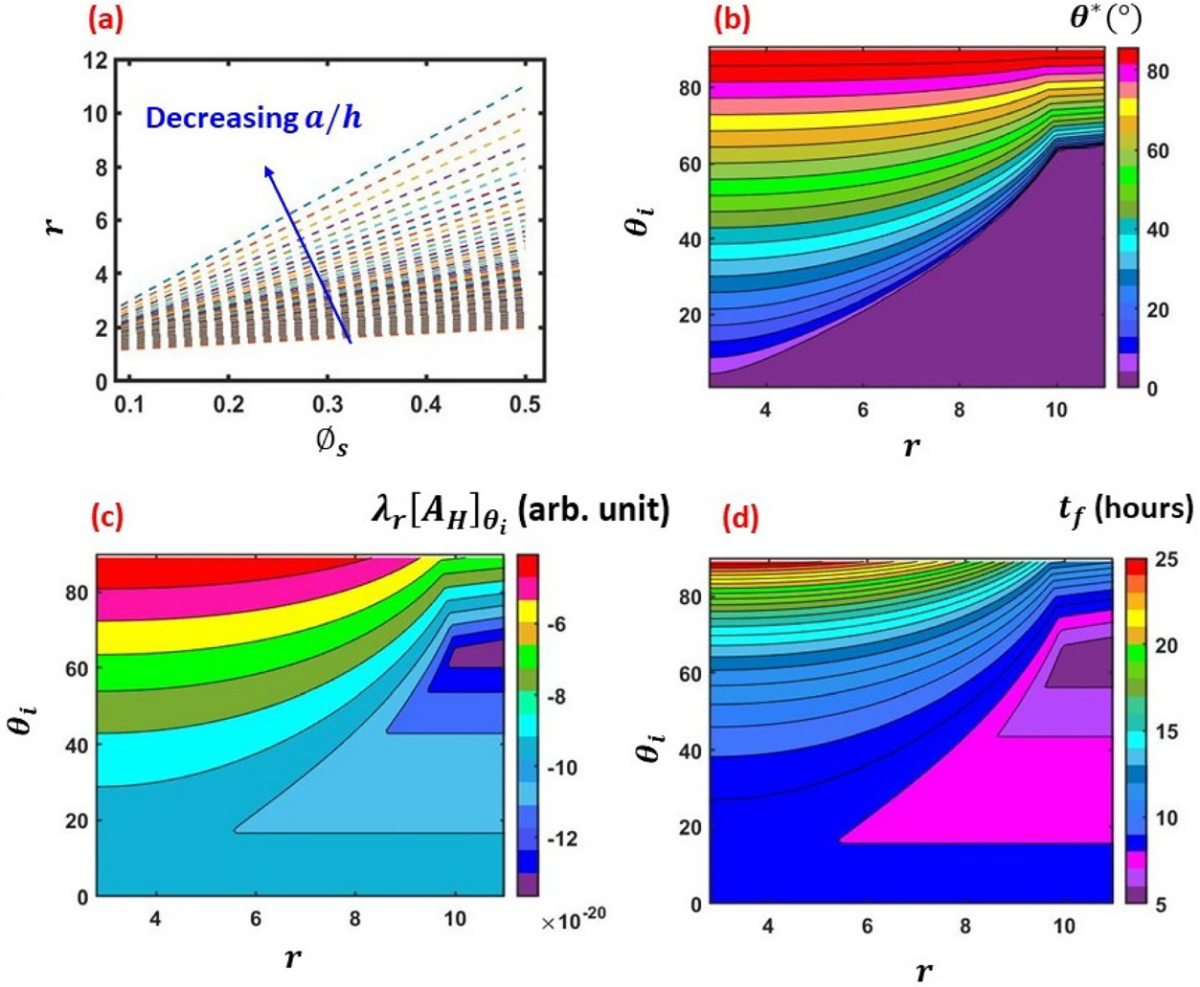
(a) Variation of *r* as a function of $\phi_{s}$ for different *a*/*h*, (b) regime map of $\theta^{*}$ with respect to *r* and *θ_i_*, (c) regime map of $\lambda_{r}A_{H}$ with respect to *r* and *θ_i_*, and (d) regime map of *t_f_* with respect to *r* and *θ_i_*. (Multimedia view) Animation depicting regime map of time varying thin-film thickness [*h*(*t*)] with respect to *r* and *θ_i_*. The results are shown for the *grooved* surfaces. Multimedia View: https://doi.org/10.1063/5.0049404.1
10.1063/5.0049404.1

Now, the thin-film lifetime (*t_f_*) for varied *θ_i_* and *r* will be analyzed for the grooved surfaces. Figure 6 depicts the results. From Fig. 6(a), it is noted that *r* varies linearly with $\phi_{s}$ and the slope increases with decreasing *a*/*h*, which is evident from Eqs. (12) and (13). In this work, *r* is chosen as the representative of roughness. Figure 6(b) depicts the regime map of $\theta^{*}$ with respect to *r* and *θ_i_*. In the context of Fig. 5(d), it is noted that for high roughness (*r *>* *10), $\theta^{*}$ approaches to zero (complete wetting) in the Wenzel regime; the Wet–Cassie regime is not reached at all. At lower roughness, an overlap between the Wenzel and Wet–Cassie regime is noted, depending upon the value of *θ_i_*. However, for low *r*, complete wetting is obtained in the Wet–Cassie regime only. This leads to a change in slope of the constant $\theta^{*}$ curves near r∼10.

Since the resultant *J*-profile *J_mod_* and hence the *h*(*t*) of the residual thin-film depends on both *r* and *θ_i_* [cf. Eq. (11)], in Fig. 6(c), a regime map of the quantity $\lambda_{r}{\left[A_{H}\right]}_{\theta_{i}}$ with respect to *r* and *θ_i_* is depicted. Importantly, $\left|\lambda_{r}{\left[A_{H}\right]}_{\theta_{i}}\right|$ is the highest ($1.1\times 10^{-19}-1.3\times 10^{-19}$) in the range r∼8−11 and $\theta_{i}\;∼\;40{}^{\circ}-70{}^{\circ}$. Hence, it is concluded that there must be having an optimum range of *r* and *θ_i_*, in which the resultant enhancement in the *J*-profile would be the highest. In Fig. 6(d) (multimedia view), we present a regime map of the thin film lifetime (*t_f_*) with respect to *r* and *θ_i_*. The associated movie presents an animation to depict the temporal evolution of thin-film thickness with time [*h*(*t*)]. The animation is presented for a total time of 27 h, with a time step of 0.5 h. The movie is played at 2 frames per second for enhanced clarity. From the results, it is noted that the optimum range of *r* and *θ_i_*, in which the highest thin-film evaporation rate is realized, is r∼5−11 and $\theta_{i}∼20{}^{\circ}-70{}^{\circ}$, which corresponds to a/h∼0.1−0.3 and a/g∼0.3−1, respectively (cf. Fig. 5). In this range, the model yields a thin-film lifetime of ∼5−8 h. Also, it is noted that at low roughness (r→1) and high *θ_i_*, the thin-film lifetime approaches to that of a smooth surface having lesser wettability (∼25 h). Hence, at low roughness (r→1), faster thin-film evaporation can be achieved by lowering *θ_i_*, and in this regime, the thin-film lifetime approaches to that of a smooth surface having higher wettability [cf. Fig. 4(b)]. This fact can be better understood from the associated animation. One can see that the thin-films in the regions of r∼5−11 and $\theta_{i}∼20{}^{\circ}-70{}^{\circ}$ dry at the earliest ($t_{f}∼5-8$ h). Thereafter, h→0 in the regions of lower roughness (r∼1.1−5) and higher wettability ($\theta_{i}∼0{}^{\circ}-30{}^{\circ}$), and in the regions of lower wettability ($\theta_{i}∼70{}^{\circ}-90{}^{\circ}$) and higher roughness (r∼9−11) with $t_{f}∼8-15$ h. The thin-film in the regions of lower wettability ($\theta_{i}∼70{}^{\circ}-90{}^{\circ}$) and lower roughness (r∼1.1−5) dries at last ($t_{f}∼15-25$ h). Figure 8(d) (multimedia view) and the associated animation, thus, demonstrate that by optimizing the *θ_i_* and *r*, five times lesser thin-film lifetime and corresponding virucidal effects can be achieved.

**FIG. 7.**
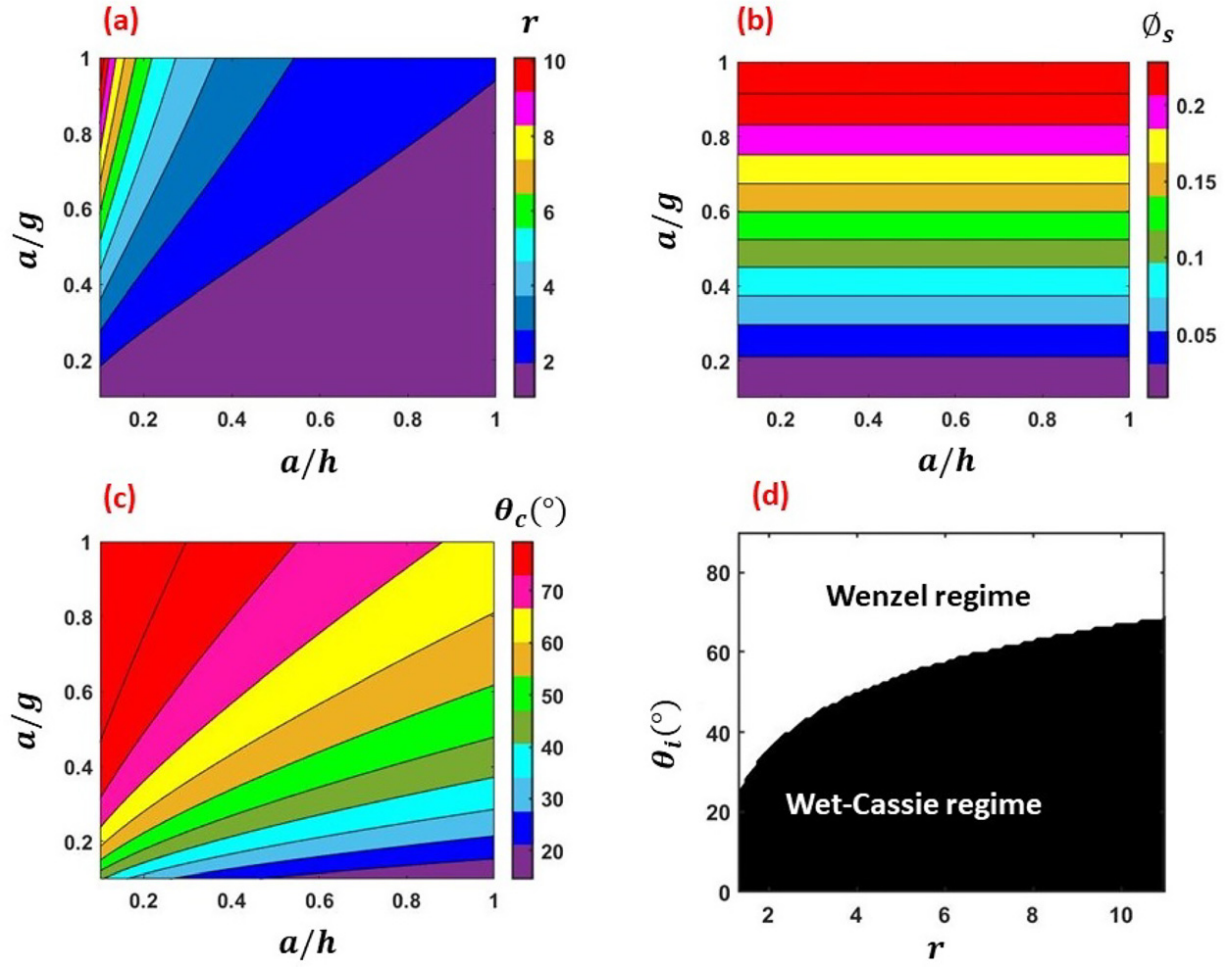
(a) Variation of *r* with respect to *a*/*h* and *a*/*g*, (b) variation of $\phi_{s}$ with respect to *a*/*h* and *a*/*g* with respect to *r* and *θ_i_*, (c) regime map of *θ_c_* for varying *a*/*h* and *a*/*g*, and (d) the Wenzel and Wet–Cassie regimes for varying *θ_i_* and *r*. The results are shown for the model *pillared* surfaces.

**FIG. 8.**
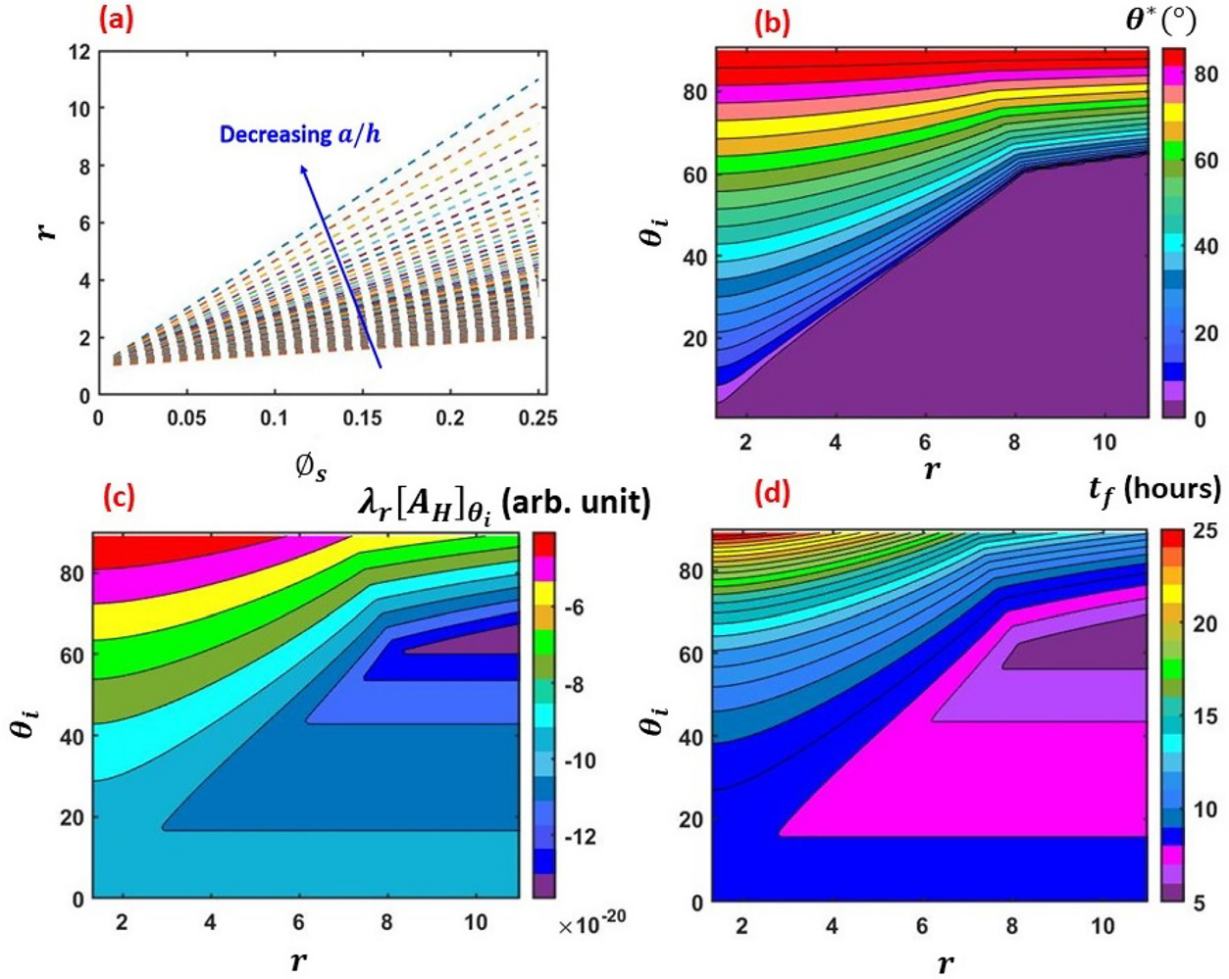
(a) Variation of *r* as a function of $\phi_{s}$ for different *a*/*h*, (b) regime map of $\theta^{*}$ with respect to *r* and *θ_i_*, (c) regime map of $\lambda_{r}A_{H}$ with respect to *r* and *θ_i_*, and (d) regime map of *t_f_* with respect to *r* and *θ_i_*. (Multimedia view) Animation depicting regime map of time varying thin-film thickness [*h*(*t*)] with respect to *r* and *θ_i_*. The results are shown for the *pillared* surfaces. Multimedia View: https://doi.org/10.1063/5.0049404.2
10.1063/5.0049404.2

Next, we analyze the model surfaces with rectangular pillars [cf. Fig. 3(b)]. Figures 7(a) and 7(b) represent the variation of *r* and $\phi_{s}$, respectively, with respect to varying *a*/*h* and *a*/*g* ratios for the model pillared surfaces. Figures 7(c) and 7(d) depict the regime map of *θ_c_* with respect to *a*/*h* and *a*/*g*, and the regimes of applicability of the Wenzel and Wet–Cassie regimes for different *r* and *θ_i_*, respectively. Noteworthy that a comparison between Fig. 5 with Fig. 7 shows that the parameters *r*, $\phi_{s}$, and *θ_c_* exhibit an universal behavior irrespective of the specific geometry (grooves/pillars). This is further manifested in the thin-film lifetime as depicted in Fig. 8. Similar to the observation for the grooved surfaces (cf. Fig. 6), for the cases of pillared surfaces also, *r* increases linearly with $\phi_{s}$ with the slope increasing as *a*/*h* decreases, which follows from Eqs. (14) and (15) [cf. Fig. 8(a)]. Furthermore, the regime map of *θ_c_* for pillared surfaces [cf. Fig. 8(b)] also exhibits similar behavior as observed for grooved surfaces [cf. Fig. 6(b)]. A comparison between Figs. 6(c) and 6(d) (multimedia view) and Figs. 8(c) and 8(d) (multimedia view) shows that the regime maps of $\lambda_{r}{\left[A_{H}\right]}_{\theta_{i}}$ and *t_f_* behave in the same way for the respective cases of grooved and pillared geometries. Similar to the case of grooved surface, for pillared surfaces also, we observe that there is a optimum condition for *r* and *θ_i_* to achieve the highest thin-film evaporation rate by virtue of the optimized $\lambda_{r}{\left[A_{H}\right]}_{\theta_{i}}$. The movie corresponding to Fig. 8(d) (multimedia view) is an animation depicting the time-varying film thickness *h*(*t*) with respect to *r* and *θ_i_*. The animation runs for a total time of 27 h with a time step of 0.5 s. For the pillared geometry, the highest thin-film evaporation rate (lowest *t_f_*) is realized for *θ_i_* within the range of 20°−70° and for *r* within the range of 3–11, which corresponds to a/h=0.1−0.3 and a/g=0.4−1 (cf. Fig. 7). Hence, the thin-film evaporation rate is dictated by the parameters *θ_i_* and *r* (or $\phi_{s}$), irrespective of the specific geometry.

## V. DISCUSSION

The generic analytical model developed herein depicts the evaporation mechanism of a thin-liquid film resting on textured surfaces having varied wettability, which aids to design surfaces with enhanced virucidal properties in the context of COVID-19. The model takes into account the effect of both surface wettability and texture within the purview of a macroscopic measurable quantity, the contact angle. The formulation allows to discern the individual contributions of chemical modification-induced altered wettability and texture-induced roughness on the evaporation mechanism of a thin-liquid film on engineered surfaces. Comparison of the model predictions with the previous virus titer measurements^50^ (cf. Sec. III) substantiates the fidelity of the theory. Thereby, the results depicted in Sec. IV for the designed surfaces are crucial in dictating the performance of the antiviral surfaces to suppress the spread of COVID-19.

First, it is deciphered that the process of fabricating antiviral surface should include a chemical treatment, so that the intrinsic wettability falls within the hydrophilic regime. This is because the physically textured surfaces should exhibit a lesser apparent contact angle than that of a smooth surface having the same chemical details in order to enhance *E_SL_*, so that the thin-film evaporation rate becomes faster by virtue of an augmented disjoining pressure (cf. Sec. IV A). Second, for a droplet deposited on an hydrophilic surface, two regimes are possible, which decide the formation of the residual thin-liquid film after the evaporation of the bulk droplet. The regimes are, as discussed in Sec. IV A, the Wenzel and the Wet–Cassie regime. The regime in which the droplet will stay is dictated both by the intrinsic wettability and surface roughness. These two regimes are characterized by different enhancement in *E_SL_*, and therefore, the resultant enhancement in the thin-film evaporation rate will be governed by both wettability and roughness. By taking into account the applicability of both the regimes, we found that there exists a optimum range of wettability (*θ_i_*) and roughness (*r*) where the thin-film lifetime is the lowest, indicating the strongest virucidal effects. Interestingly, as demonstrated in Sec. IV C in light of the comparison between Figs. 6 (multimedia view) and 8 (multimedia view), the evaporation dynamics of the thin-film is dictated by *r* and *θ_i_* irrespective of the geometric details (grooved/pillared). This is also true for the optimization of parameters. Furthermore, it is worth noting that both for the cases of grooved and pillared geometry, for $\theta^{*}∼17{}^{\circ}$ and r∼1.3, the thin-film lifetime returns in ∼8 h. This is also consistent with the titer decay timescale reported by Hasan *et al.*,^50^ wherein the coronavirus survival time was found to be ∼6 h on surfaces decorated with nanostructures grouped in ridges, wherein $\theta^{*}=17.7{}^{\circ}$ and r∼1.24 (area %∼23.8). Hence, the generic model developed herein, by taking into account the effect of wettability and roughness, rightly captures the essential mechanism behind the virucidal properties of textured surfaces. It demonstrates that the optimum thin-film evaporation rate can be achieved by tailoring *r* and *θ_i_*, irrespective of the specific geometry of the texture. This is essentially the consequence of Eq. (6), wherein all the modification induced by the surface engineering process has been accommodated within the ambit of contact angle.

The present analysis expands the applicability of the process; one may fabricate any kind of geometric structures as per the convenience and the availability of the fabrication technique in order to achieve the same outcome. The optimized operating conditions reported in Figs. 6 and 8 should be accounted for to obtain the best results. We also point out that the findings reveal that within the preferred range of *θ_i_*, there exists a range of roughness (r∼5−11) for which the optimized thin-film evaporation rate (and hence the virucidal effect) is realized. This is further beneficial because surface texturing involves sophisticated techniques such as laser writer, electron beam lithography, focused ion beams, and chemical etching. Our results indicate that for the given range of *θ_i_*, one may choose any *r* within the range of optimized thin-film evaporation rate, thereby optimizing the operating time and cost. Hence, surface texturing and tailoring wettability can be considered as a viable tool for inducing enhanced virucidal properties to surfaces.

**FIG. 9.**
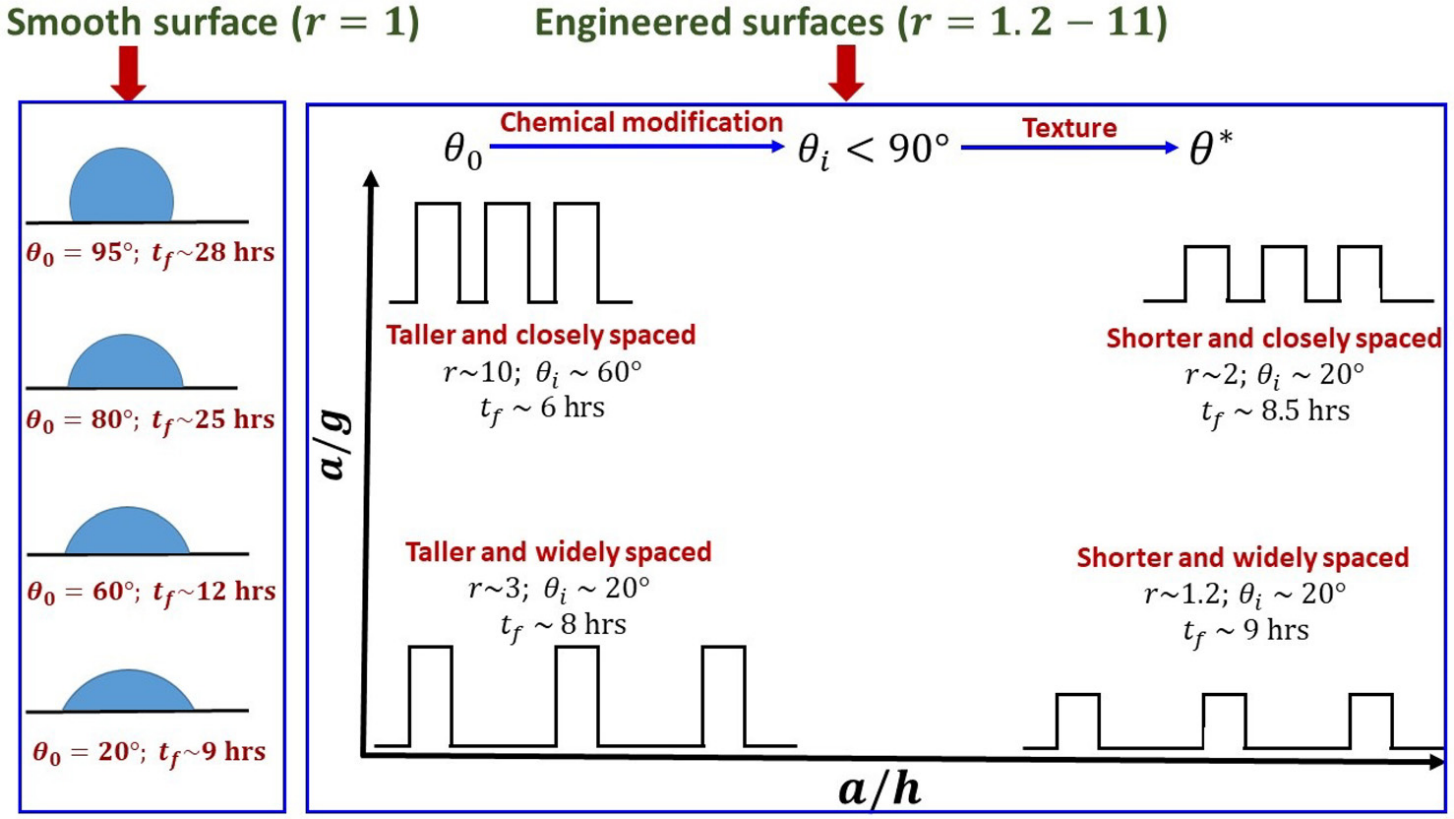
A case study of the virucidal properties across different surface wettability and texture, shown schematically (not to scale). The values depicted for *r*, $\theta^{*}$, *θ_i_*, and *t_f_* are representative, extracted from the data shown in Figs. 4–8, for the purpose of demonstration of different regimes with respect to surface wettability and texture, which lead to optimized virucidal effects.

For the sake of better clarity and contrast, we present a case study of the thin-film lifetime or the virucidal effects across varying wettability and texture. Figure 9 schematically represents the same, which is an excerpt of the findings presented in this communication. As outlined in Sec. IV B, it is plausible to represent rough surface by a square-wave pattern in the two-dimensions.^69^ For a given surface, if $\theta_{0}>90{}^{\circ}$, the surface engineering process should include a chemical modification by which $\theta_{i}<90{}^{\circ}$ is obtained in order to enhance the thin-film evaporation rate, and thereby the virucidal effects by introducing additional roughness. Furthermore, surfaces with taller and closely packed surface heights (say, for example a/h∼0.1 and a/g∼0.9) fall within the aforementioned range required for optimization with respect to roughness (r∼10), and therefore returns the least thin-film lifetime ($t_{f}∼6$ h) if $\theta_{i}<90{}^{\circ}$ falls within the aforementioned range required for optimization with respect to intrinsic wettability (say, for example, $\theta_{i}=60{}^{\circ}$). At lower roughness (r∼1−3), the thin-film lifetime of the engineered surfaces can be minimized by lowering *θ_i_* (say, for example, 20 °). The thin-film lifetime in this limit approaches to that of smooth surfaces having higher wettability ($\theta_{0}∼20{}^{\circ}$).

It is important to mention here that although the Wenzel state is characterized by a higher enhancement in the hydrophilicity, or a higher enhancement in *E_SL_*,^63,64^ for a given surface roughness (given *r*), the least thin-film lifetime is always returned for a *θ_i_* falling in the Wet–Cassie regime (cf. Figs. 5–9). This is because, in the present analysis, both the effect of roughness and wettability have been considered by virtue of Eq. (11). The absolute value of the product $\lambda_{r}{\left[A_{H}\right]}_{\theta_{i}}$ is the deciding factor for the resultant evaporation dynamics of a thin-film on an engineered surface. It can be seen from Figs. 6(c) and 6(d) (multimedia view) and 8(c) and 8(d) (multimedia view) that $\left|\lambda_{r}{\left[A_{H}\right]}_{\theta_{i}}\right|$ is always the highest for *θ_i_* values within the Wet–Cassie regime for all roughness considered in the present study. This is essentially because the Wet–Cassie regime inherently falls in the regions of higher wettability, i.e., higher surfaces free energy of the underlying solid, which automatically leads to a higher excess energy/disjoining pressure within the thin-liquid film.

It is worth mentioning that a textured surface can also have hierarchical features, i.e., may contain additional tiers.^69^ As demonstrated by Frankiewicz and Attinger,^69^ the total roughness factor *r_tot_* for a surface with *n* tiers can be represented by $r_{\mathrm{tot}}=\prod_{i=1}^{n}r_{i}$, where *r_i_* is the roughness factor due to *i*th tier. Hence, for surfaces having multiple tiers, *r* should be replaced as *r_tot_* in the above analyses. It is quite straightforward to show that for the same *a*/*h* and *a*/*g* ratios across all tiers, adding tiers would further accelerate the thin-film evaporation. Therefore, with the optimized conditions with respect to *θ_i_*, *a*/*h*, and *a*/*g* found in the present model, adding tiers would further make the surface more and more antiviral.

A few limitations of this study are discussed, which can be addressed in the future. First of all, we have considered a surrogate droplet of pure water and the corresponding residual thin-liquid film in the present analysis. Real respiratory droplets or saliva may contain biological solutes whose drying has been explained by the Raoult's effect.^72^ Yet, the error for these approximations considered in the present study is within ∼25%.^45,72^ The shear stress associated with the presence of the virus is also negligible.^44^

Finally, we discuss the relevance of the present findings in the context of molecular surface effects. The developed model analyses the dynamics of the liquid thin-film on engineered surfaces on the basis of the disjoining pressure within the film by accounting the solid–liquid adhesive interaction [cf. Eqs. (4), (5), and (7)]. Adhesion has a molecular origin; the contact angle is determined by the interplay between the cohesive and adhesive intermolecular interaction between the liquid and solid in question.^51,67,73^ The adhesive energy determines the threshold static friction that needs to be overcome to commence a motion of the triple phase contact line against a solid surface.^74^ Moreover, recent molecular dynamics studies^75–77^ on liquid nanoflows confined within solid walls have disclosed that the flow pattern is distorted by roughness, which generates a viscosity gradient and modifies the velocity profile near the solid wall. The above-mentioned phenomena in the presence of varying wettability and texture of the walls can be studied within the ambit of the present formulation, which could be a future scope of research.

## VI. CONCLUSIONS

In closure, we have explored the combined effect of varying surface wettability and texture on the virucidal properties of surfaces in the context of COVID-19. We propose design of antiviral surfaces, which could help reducing the survival of coronavirus on impermeable surfaces, thereby mitigating the spread of COVID-19 via fomite route. Previously, it was reported that the lifetime of a residual thin-film after the diffusion limited evaporation of a respiratory droplet is correlated with the coronavirus survival time. Therefore, we analyze the said virucidal properties by modeling the evaporation mechanism of the thin-film on textured surfaces with varied wettability and roughness. The generic model developed herein could explain the earlier virus titer measurements on textured surfaces with reasonable fidelity. Thereafter, model surfaces having parallel rectangular grooves and rectangular pillars have been analyzed on the basis of the model. It has been found that the thin-film evaporation rate is a function of the roughness factor and the intrinsic contact angle, irrespective of the specific geometry considered. Also, the optimum range for the intrinsic wettability and roughness, for which the fastest thin-film evaporation rate is obtained to yield the most conducive virucidal effects, has been disseminated in the present communication. The findings are useful for fabricating surfaces with virucidal properties of surfaces, especially applicable to medical and pathological laboratory equipment, thereby mitigating the spread of COVID-19 from these sources.

## Data Availability

The data that support the findings of this study are available from the corresponding author upon reasonable request.
